# Mutation E87Q of the β1-subunit impairs the maturation of the cardiac voltage-dependent sodium channel

**DOI:** 10.1038/s41598-017-10645-y

**Published:** 2017-09-06

**Authors:** Debora Baroni, Cristiana Picco, Oscar Moran

**Affiliations:** istituto di Biofisica - CNR, via De Marini 6, 16149 Genova, Italy

## Abstract

Voltage-dependent sodium channels are responsible of the rising phase of the action potential in excitable cells. These membrane integral proteins are composed by a pore-forming α-subunit, and one or more auxiliary β subunits. Mutation E87Q of the β1 subunit is correlated with Brugada syndrome, a genetic disease characterised by ventricular fibrillation, right precordial ST segment elevation on ECG and sudden cardiac death. Heterologous expression of E87Q-β1 subunit in CHO cells determines a reduced sodium channel functional expression. The effect the E87Q mutation of the β1 subunit on sodium currents and α protein expression is correlated with a reduced availability of the mature form of the α subunit in the plasma membrane. This finding offers a new target for the treatment of the Brugada syndrome, based on protein maturation management. This work highlights the role played by the β1 subunit in the maturation and expression of the entire sodium channel complex and underlines how the defective interaction between the sodium channel constituents could lead to a disabling pathological condition.

## Introduction

Voltage gated sodium channels (NaChs) open and close on a millisecond time scale in response to changes in cell membrane potential. This activation-inactivation cycle mediates the transient influx of sodium ions that generates the action potential in most types of excitable cells^1^. Native NaChs are composed by one larger pore-forming α subunit and one or two smaller auxiliary β subunits^2, 3^. Heterologous expression studies either in *Xenopus* oocytes or mammalian cells have disclosed the functional roles of Nach α and β subunits. The α subunit forms the ion pore and contains the domains that confer the voltage-depedent gating and the pharmacological properties of the channel^2^. The β subunits modulate channel kinetics and voltage dependence^4–7^, regulate cell surface channel expression^8–11^ and contribute to cell-cell and cell-matrix adhesion, participating in cellular aggregation, ankyrin recruitment, and neurite outgrowth^12–18^. Nine different voltage-gated α subunits designated Nav1.1–Nav1.9, and a related, non-voltage-gated atypical isoform named Navx, have been found in mammals, each encoded by a different gene^19^. These genes produce polypeptides with a high degree of sequence identity, but a distinctive tissue specificity expression for a review see ref. 20. The isoform Nav1.5 is expressed predominantly in the heart.

Four accessory β subunits (β1–4), and a splice variant of β1 designated as β1A/B, have been identified in mammals. They are encoded by four different genes (SCN1B-4B)^19^, and all of them belong to the immunoglobulin (Ig) super-family of cell adhesion molecules (CAM). The highly conserved extracellular Ig motif is stabilised by an intra-chain disulphide bridge^21, 22^ and may interact with different cytoskeleton and extracellular matrix proteins^12, 23, 24^. The closely related β1 and β3 subunits (~45% sequence identity) are no-covalently associated with the α subunits, whereas the β2 and the β4 subunits (35% identity) are linked through a disulphide bond to the α subunits^25^. NaCh β subunits are all detectable in brain tissues, peripheral nerves, heart, and skeletal muscle.

NaChs are major pharmacological targets in local anaesthesia, cardiac arrhythmia, analgesia, epilepsy and bipolar disorder, and have also been investigated in conditions such as stroke, myotonia and Parkinson’s disease^26^. An increasing number of inherited disorders have been associated with NaCh mutations including skeletal muscle diseases, cardiac disorders, and epilepsy^27, 28^. Focusing on β1 subunit, mutations in this subunit cause inherited diseases that selectively affect the central nervous system or the heart, such as generalized epilepsy with febrile seizure plus type 1 (GEFS+), the Brugada syndrome 5 and cardiac conduction diseases [for a review see ref. 9].

The β1 mutation E87Q is linked to the Brugada syndrome 5^9, 29^. This mutation is due to a 259G-C transversion in exon 3 of the human SCN1B gene, that determines a substitution of a highly conserved glutamic acid with a glutamine at position 87 within the Ig-loop, which is common to both the β1 and β1A/B transcripts. Functional studies of the E87Q mutation show that, while the co-expression of the cardiac Nav1.5 NaCh subunit with the wild-type (WT-) β1 in mammalian cells significantly increases sodium current density, the co-expression with the mutant E87Q-β1 does not increase the sodium current with respect to WT-β1. In addition, WT-β1 produces negative shifts in the voltage dependence of Nav1.5 activation and inactivation, whereas mutant E87Q-β1 shifts only the voltage dependence of inactivation^29^.

Here, we studied the regulation of the expression of the Nav1.5 NaCh subunit by WT- and E87Q-β1 subunits. We pointed out that the effect of the E87Q mutation of the β1 subunit on sodium current and Nav1.5 protein expression is correlated with a reduction of mature Nav1.5 α subunit availability in the cell membrane. This finding offers a new target for the treatment of the Brugada syndrome, based on a protein maturation management.

## Results

### Functional expression of sodium channels in CHO transfected cells

Expression of Nav1.5, either alone or co-expressed with WT- and E87Q-β1 subunit, resulted in well characteristic sodium currents. The amplitude of the sodium current increased up to 2–3 fold during the first 10 min of the experiment, and achieved a stable level after 15–20 min. Therefore, all measurements were done after 20 min from the beginning of the experiment. To evaluate the functional expression of NaCh, the peak amplitude of the maximal sodium current was normalized to cell capacitance in order to obtain the sodium current density.

**Figure 1:**
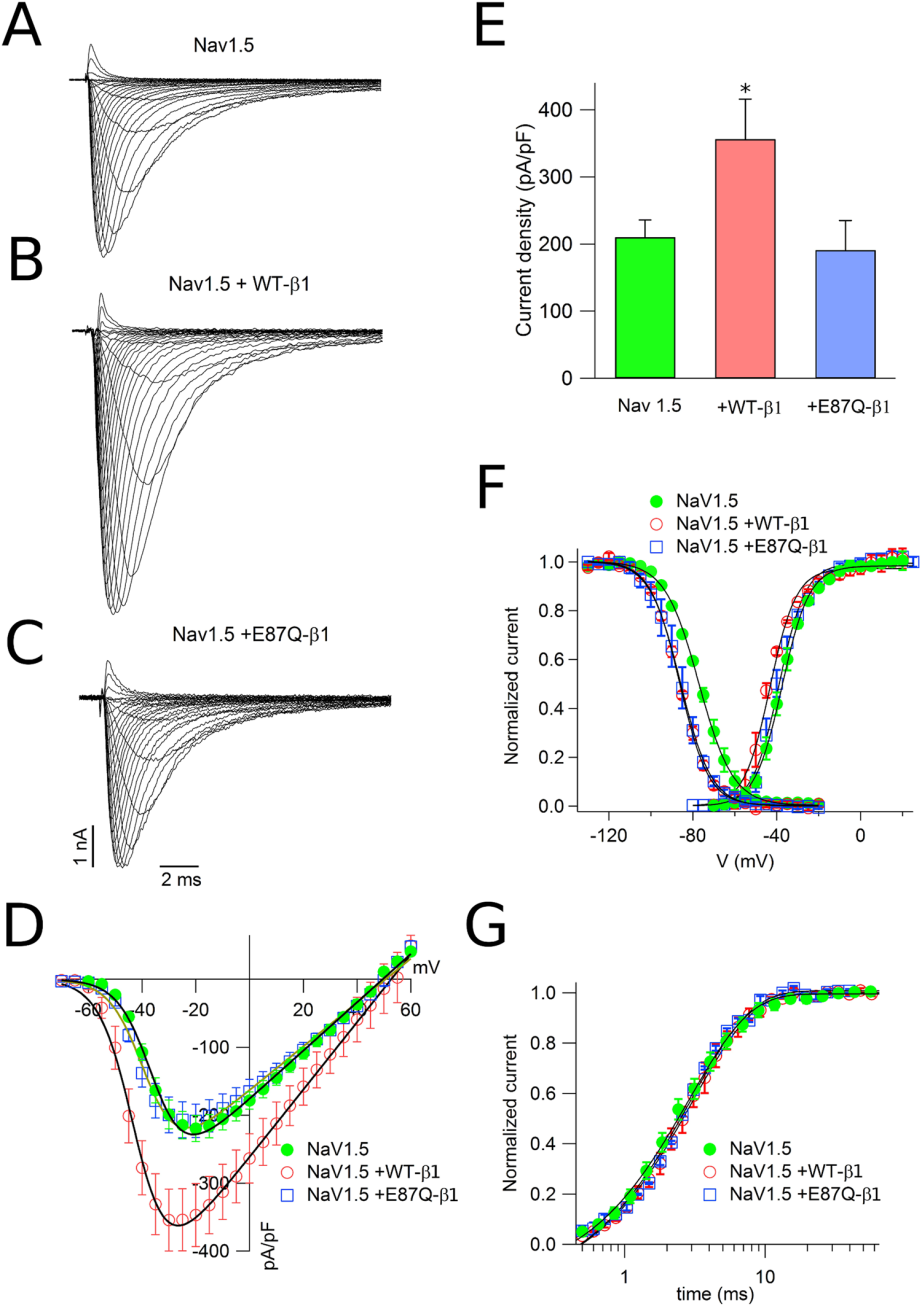
Functional expression of sodium channel in CHO cells. Comparison of families of whole cell currents elicited by depolarizations from −70 to + 60 mV in CHO cells transfected with Nav1.5 (**A**), Nav1.5 + WT-β1 (**B**), and Nav1.5 + E87Q-β1 (**C**). (**D**) The mean of the peak current amplitudes for the three experimental conditions are normalized to cell capacity and plotted as function of membrane voltage. Data are fitted to eq. 1 Currents evoked by CHO cells expressing Nav1.5 are represented with filled circles, those by Nav1.5 + WT-β1 with empty circles, and those by Nav1.5 and E87Q-β1 with empty squares. The bars in (**E)** represent the average ( ± s.e.m.) of the peak current density evoked by a depolarizing pulse of −25 mV; the asterisk (*) indicates a significant difference (*p < *0.05) in the comparison with Nav1.5 transfected cells. (**F**) The voltage dependence of steady-state activation and inactivation (curves of CHO cells expressing Nav1.5 (filled circles), Nav1.5 and WT-β1 (empty circles), and Nav1.5 and E87Q-β1 (empty squares). Continuous lines represent the best fit of the activation curve with eq. 1, and the inactivation curve with eq. 2. Recovery from inactivation at a holding potential of −120 mV in all examined conditions is represented in (**G**) Continuous lines stand for the best fit of the data with a single exponential curve.

Figure 1A shows traces of Nav1.5 sodium currents evoked by 20 ms depolarising pulses from a holding potential of −120 mV to a test potential from −70 to + 60 mV in 5 mV increments. Co-expression of Nav1.5 and WT-β1 (Fig. 1B and D) significantly increased sodium current over Nav1.5 alone, while currents recorded with Nav1.5 co-expressed with E87Q-β1 (Fig. 1C and D) were not different from Nav1.5 alone. Figure 1E summarizes the mean current densities of Nav1.5 and Nav1.5 co-expressed with WT- and E87Q-β1 subunits. WT-β1 produced negative shifts in the voltage dependence of Nav1.5 activation and inactivation while E87Q-β1 shifted the voltage dependence of inactivation to negative potentials (similar to WT-β1) but did not alter the voltage dependence of activation (Fig. 1F and Table 1). Co-expression of WT- or mutant β1 with Nav1.5 did not alter recovery from inactivation (Fig. 1G and Table 1). Our results confirm that mutation E87Q abolishes the increment of functional expression of NaCh currents induced by the expression of the WT-β1subunit, as well as the shift of the activation curve, as previously reported^29^.

**Table 1:** *Functional parameters of sodium currents recorded in Nav1.5, Nav1.5* + *WT−, and Nav1.5* + *E87Q-β1 CHO transfected cells*.

	***V***_**1/2**_ **(mV)**	***S***_***a***_	**n**	***V***_***h***_ **(mV)**	***S***_***h***_	**n**	**τ @ −120 mV (ms)**	**n**
Nav1.5	−36 ± 2	6.4 ± 0.3	13	−77 ± 2	8.3 ± 0.4	12	2.9 ± 0.3	11
Nav1.5/WT-β1	−43 ± 2(*)	6.7 ± 0.4	16	−85 ± 2 (**)	8.3 ± 0.4	13	3.3 ± 0.4	11
Nav1.5/E87Q-β1	−38 ± 2	6.3 ± 0.4	11	−83 ± 2 (**)	8.0 ± 0.4	9	3.0 ± 0.4	9

*V*_1/2_ is the half activation potential, *S*_*a*_ is the maximum exponential slope of the function, respectively. *V*_*h*_ is the half inactivation potential and *s*_*h*_ is the voltage dependence of inactivation. τ is the time constant for the recovery from inactivation, measured at 120 mV. A number of at least 3 independent transfections has been performed. For each condition measured the number of cells used is indicated as n. Data are expressed as mean ± standard error (sem). Comparisons among current densities were performed with the Student’s t test. Asterisks (*) and (**) indicate a significant difference (p < 0.05 or p < 0.01) in the comparison with CHO cells transfected with Nav1.5 alone.

### Expression of sodium channel proteins

**Figure 2:**
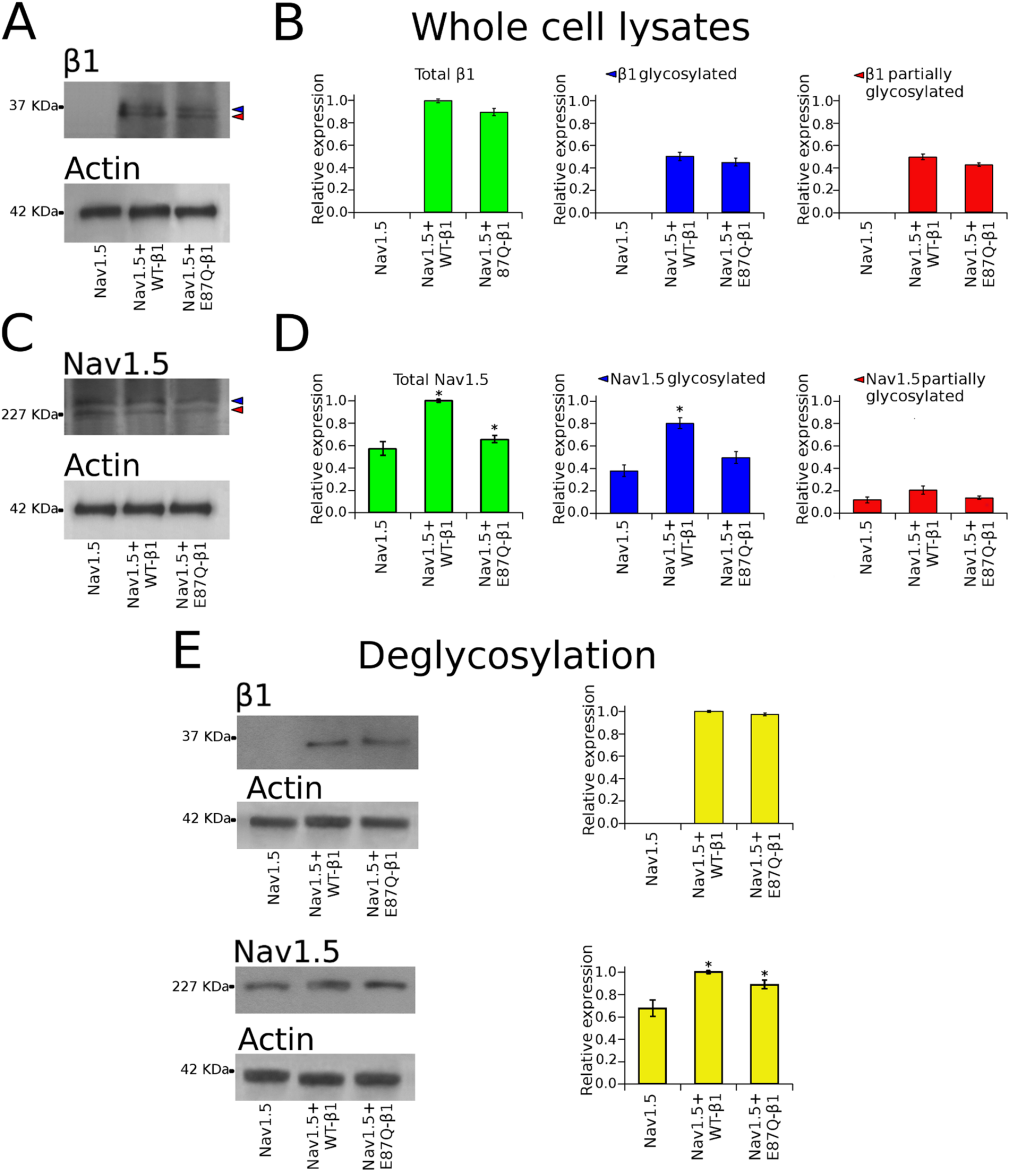
Expression of sodium channel β1 and Nav1.5 proteins in CHO cells. (**A**) Western blots images of the expression of β1 (at the top) and control actin bands (at the bottom) detected on the same blotting membrane. (**C**) western blot images representing the expression of Nav1.5 (at the top) and the corresponding control actin bands (at the bottom). In each image, the left lane corresponds to the total lysates of cells transfected with sole Nav1.5, the central lane to cells transfected with Nav1.5 + WT-β1, and the right lane to Nav1.5 + E87Q-β1. In both cases, there are two bands one of a higher molecular weight, corresponding to the fully glycosylated, mature form, of the proteins (red head arrows), and another of lower molecular weight, corresponding to the partially or unglycosylated, immature form, of the proteins (blue head arrows). The relative expression of total, fully glycosylated and partially glycosylated fractions of the β1 subunit are shown in (**B)**. The relative expression of total, fully glycosylated and partially glycosylated fractions of Nav1.5 α subunit are shown in (**D)**. Immunoblots showing the effect of the treatment with the de-glycosylation enzymes are shown in (**E)**. Digested NaCh protein samples are revealed as a unique immunoreactive band. Actin has been used as control (at the bottom of the panels). The relative expression of digested β1 and Nav1.5 NaCh protein samples is shown in the left panels. Bars represent the mean and the standard error of the mean of data estimated from at least three independent experiments. The intensity of each band was scaled to the intensity of the band corresponding to the actin detected in the stripped blotting membrane, and successively was normalised to the average expression level of total β1 or total Nav1.5 in Nav1.5 + WT-β1 samples. Asterisks (*) indicate a significant difference (*p < *0.05) of the expression level of β1 or Nav1.5 NaCh subunits in Nav1.5 + WT-β1 or Nav1.5 + E87Q-β1 transfected cells in comparison with cells transfecetd with Nav1.5 alone.

Western blot images revealing the expression of Nav1.5, WT- and E87Q-β1 subunits in total lysates of Nav1.5, Nav 1.5 + WT-β1 and Nav1.5 + E87Q-β1 CHO transfected cells are shown in Fig. 2A and C respectively. β1 or Nav1.5 NaCh subunits are revealed as two electrophoretic bands of slightly different molecular weights (red and blue head arrows in Fig. 2A and C). These bands may correspond to different glycosylation states of the NaCh subunits^30^. Control experiments showed that antibodies against β1 or Nav1.5 did not reveal the presence of these NaCh subunits in CHO untransfected cells. The removal of both N- and O-linked glycans from both NaCh subunits resulted in a single band corresponding to the β1 and Nav1.5 unglycosylated isoforms, respectively (Fig. 2E). Accordingly, the higher molecular weight bands were referred as corresponding to the fully glycosylated forms of β1 and Nav1.5 proteins, respectively. The lower molecular weight bands, of ~37 and ~227 KDa, were considered as NaCh partially or unglycosylated forms. The quantification of total β1 or Nav1.5 subunit expression was done taking into account both bands.

The expression of β1 subunit protein in total lysates was very similar in cells transfected with Nav1.5 + WT-β1 and with Nav1.5 + E87Q-β1 (Fig. 2B, left panel). The separated quantification of the fully-glycosylated, and partially or unglycosylated forms of WT- and E87Q-β1 (Fig. 2B, central and right panels, respectively) shows that the expression of the two forms is also very similar in both cases. Conversely, important differences were found in the expression of Nav1.5 in total lysates when cells were co-transfected with WT-β1 and E87Q-β1. Expression of the WT-β1 subunit determined an increase of 1.8 fold of total Nav1.5 protein expression, in comparison to Nav1.5 alone (Fig. 2D, left panel). Conversely, the co-transfection of the mutant E87Q-β1 determined a significant lesser increase (1.2 fold) of the expression of total Nav1.5 protein (Fig. 2D, left panel), suggesting that the efficiency of the β1 subunit to enhance the expression of the α subunit is somehow compromised by the mutation.

Interestingly, WT-β1 and mutant E87Q-β1 subunits have a different effect on the expression of the fully glycosylated and unglycosylated forms of the Nav1.5 protein. Indeed, the co-transfection of the WT-β1 subunit determined a 2-fold increase of the fully-glycosylated fraction of the Nav1.5 subunit. In contrast, co-transfection with the mutant E87Q-β1 subunit did not increase the level of the glycosylated fraction of Nav1.5 protein (Fig. 2D, central panel). The average expression level of the unglycosylated form the Nav1.5 protein did not change by the co-expression of WT-β1 or E87Q-β1 (Fig. 2D, right panel).

**Figure 3:**
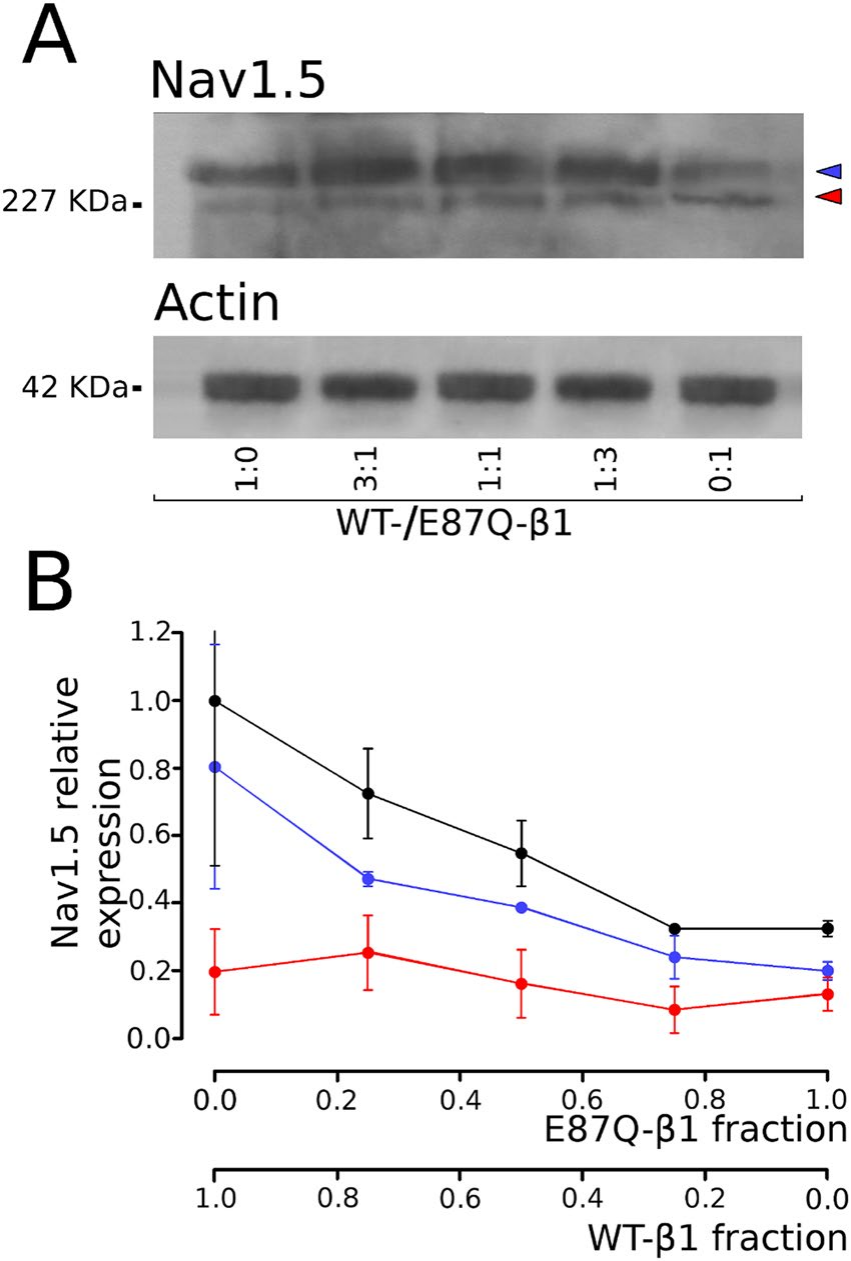
Comparison of Nav1.5 expression in total lysates of cells co-trasfected with Nav1.5 and different molar ratios of WT- and E87Q-β1 subunit cDNAs. (**A**) Western blot image of lysates of cells co-transfected with Nav1.5 and variable WT-β1:E87Q-β1subunit molar ratios, as indicated. Actin bands detected on the same blotting membranes are shown below. **(B)** Normalised values of the expression of total (black), mature, fully glycosylated (blue), and immature, partially or unglycosylated (red) Nav1.5 are plotted against the corresponding co-transfected β1 subunit fractions. Bars represent the mean and the standard error of the mean of data estimated from at least three independent experiments.

**Figure 4:**
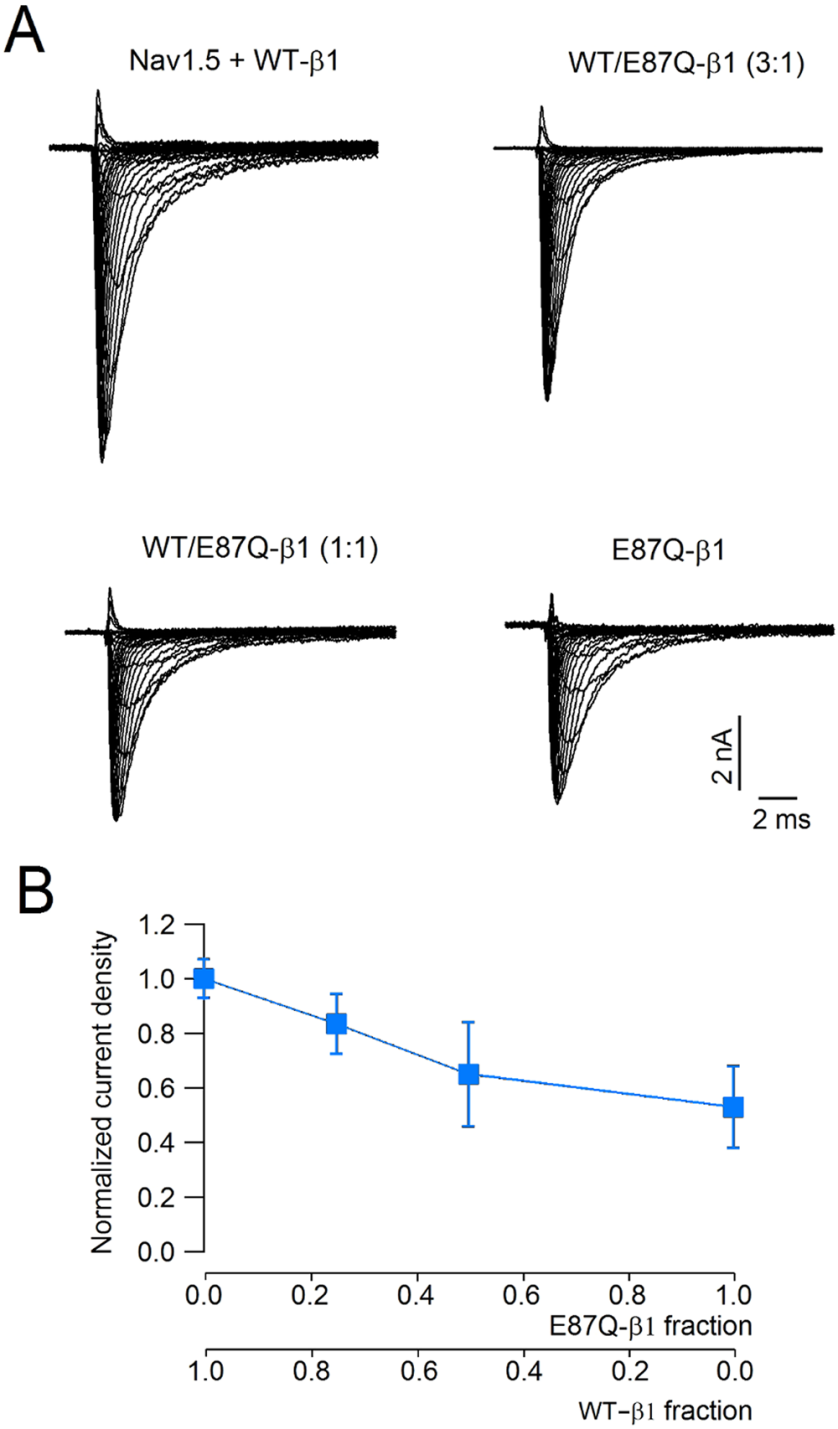
Functional expression of CHO cells co-trasfected with Nav1.5 and different molar ratios of WT- and E87Q-β1 subunit cDNAs. (**A**) Comparison of families of whole cell currents elicited by depolarizations from −70 to + 60 mV in CHO cells transfected with Nav1.5 and variable WT-β1:E87Q-β1subunit molar ratios, as indicated. (**B**) The normalized current density is plotted versus the different ratios of WT and E87Q. Bars represent the mean and the standard error of the mean of data estimated from at least ten independent experiments.

To test the correlation between the expression magnitude of the glycosylated fractions of NaCh α and β1 subunits, Nav1.5 was co-expressed with variable molar ratios of WT- and mutant E87Q-β1 cDNAs, and the expression of the glycosylated and unglycosylated fractions were determined for each β1 combination (Fig. 3). Consistently, the expression of total Nav1.5 protein progressively decreased when Nav1.5 cDNA was co-transfected with decreasing molar concentrations of WT- and increasing molar concentrations of E87Q-β1 subunits (black in Fig. 3B). The evaluation of the two Nav1.5 bands separately, showed that the expression of the fully-glycosylated fraction of Nav1.5 subunit is well correlated with the amount of co-transfected WT-β1, and decreased when the molar ratio of co-transfected WT-/E87Q-β1 cDNAs decreased (blue in Fig. 3B). In contrast, the expression level of the partially or unglycosylated form the Nav1.5 protein remained unchanged, independently from the molar ratio of the WT- and mutated β1 subunits (red in Fig. 3B). Consistently, the co-expression of Nav1.5 with variable molar ratios of WT- and mutant β1 cDNAs resulted in a smaller current density as the fraction of the E87Q- β1 mutant increases (Fig. 4).

### Cell surface expression of sodium channels

**Figure 5:**
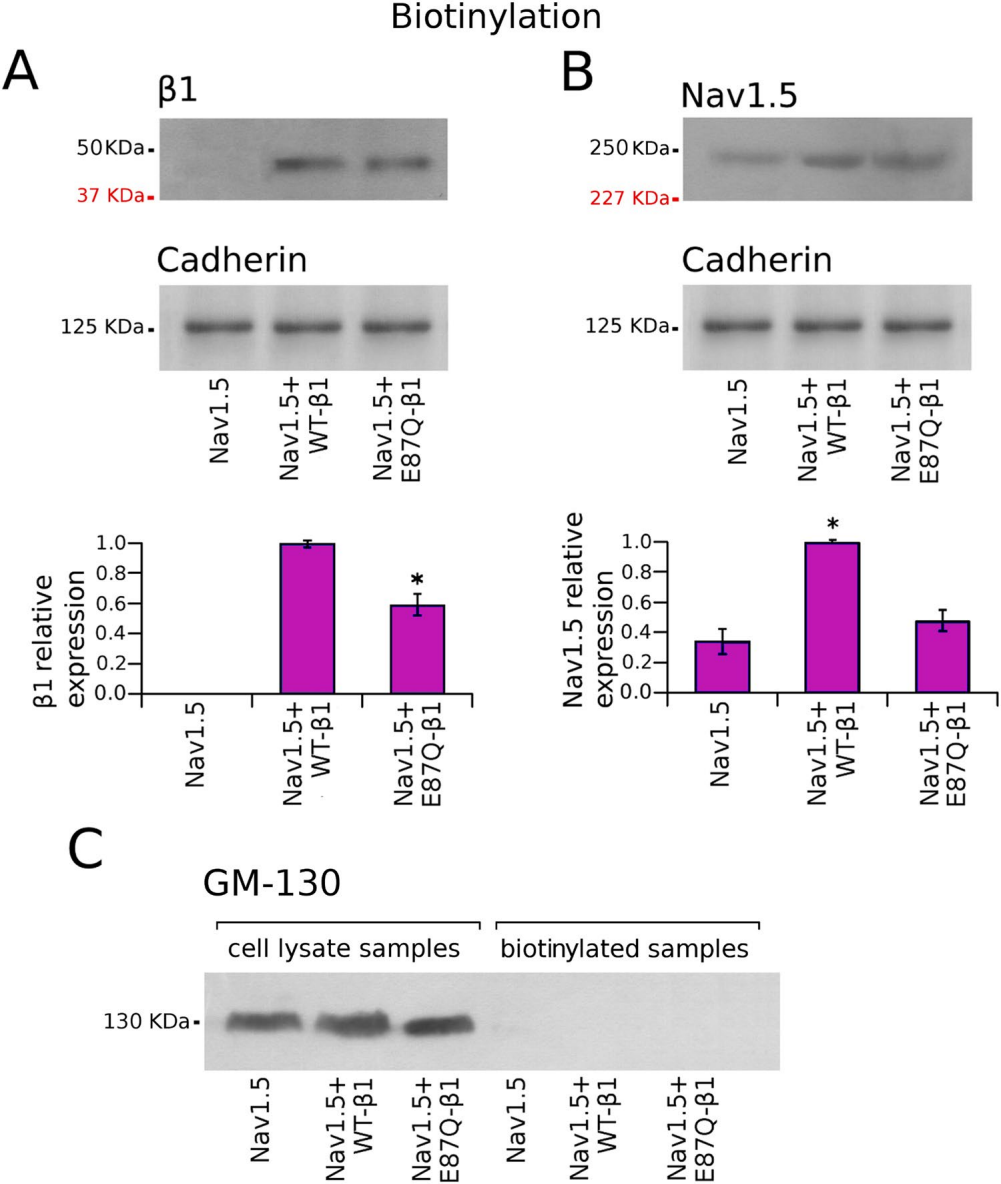
Cell surface expression of sodium channel β1 and Nav1.5 proteins in CHO cells. Western blots of the biotinylated fractions of β1 (**A**) and Nav1.5 (**C**) in cells transfected with Nav1.5 alone, Nav1.5 + WT-β1 and Nav1.5 + E87Q-β1. On the left of the blots are shown the the molecular weight of the marker proteins used during SDS-PAGE electrophoresis (in black) and the molecular weight of native, immature β1 and Nav1.5 (in red). The plasma membrane marker cadherin, revealed on the same blotting membranes, has been used as control (at the bottom of each panel). Bar graphics showing the relative expression of β1 and Nav1.5 subunits is displayed at the bottom of panels A and B, respectively. The intensity of each band was scaled to the intensity of the band corresponding to the cadherin detected in the stripped blotting membrane and was normalised to the average expression level of total β1 or total Nav1.5 in Nav1.5 + WT-β1 samples. Bars represent the mean and the s.e.m. of data calculated from at least 3 independent experiments for each condition. Asterisks (*) in (**A**) indicate a significant difference (*p* < 0.05) of β1 protein cell surface expression level in Nav1.5 + E87Q-β1 transfected cells in the comparison with Nav1.5 + WT-β1 transfected cells. Asterisks (*) in (**B**) indicate a significant difference (*p* < 0.05) of Nav1.5 protein cell surface expression level in Nav1.5 + WT-β1 or Nav1.5 + E87Q-β1transfected cells in comparison with cells transfected with Nav1.5 alone. (**C**) Anti-GM130 primary antibody was used as negative control to show that biotinylation revealed the specific labelling of only those NaCh proteins expressed on the cell membrane.

Cell surface biotinylation experiments were done to assess whether the expression of the glycosylated Nav1.5 α subunit depended on the presence of the β1 subunit on the plasma membrane (Fig. 5). Data showed that the expression of biotinylated mutant E87Q-β1 was reduced to 60% respect to the expression of WT-β1 (Fig. 5A, lower panel). Western blot quantification of the biotinylated Nav1.5 α subunit shows that co-expression of WT-β1 induced a 2.9-fold increase of the α subunit expression on the cell surface, compared with cells transfected with Nav1.5 alone (Fig. 5B, lower panel). In contrast, the Nav1.5 cell surface expression was not significantly modified in cells co-transfected with Nav1.5 + E87Q-β1 (Fig. 5B, lower panel). In order to ensure that the biotinylation reflects the specific labelling of proteins expressed on the cell surface, immunoblots were also probed with anti-GM130 primary antibody, which specifically binds to a Golgi protein. As expected, no GM130 signal was detected in NaCh biotinylated fractions compared to positive immunoblot detection observed in total lysate samples (Fig. 5C).

**Figure 6:**
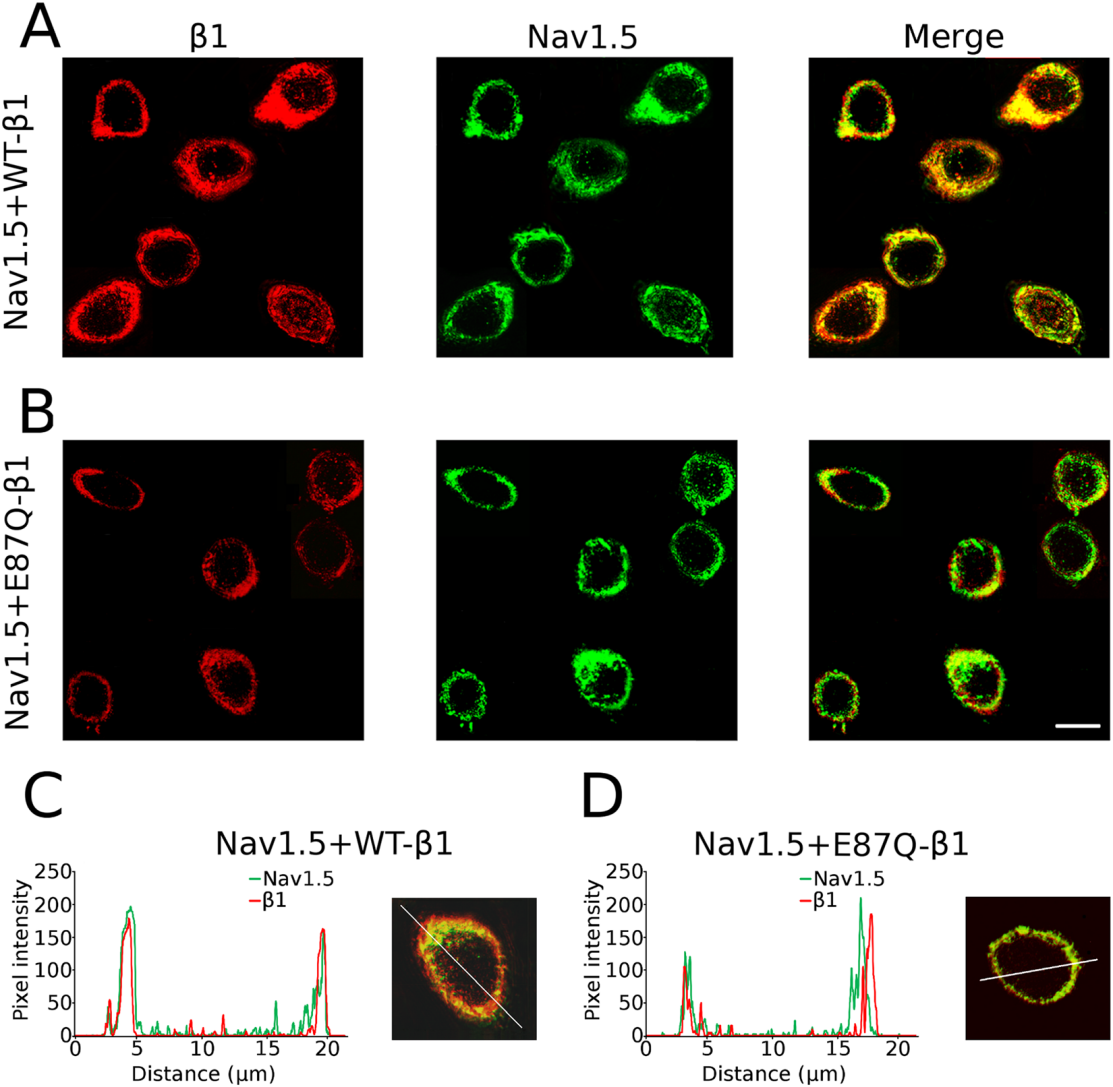
Cellular localization of β1 and Nav1.5 α subunits. Representative images showing immunofluorescence of β1 subunit (red) and Nav1.5 α subunit (green) in cells co-transfected with Nav1.5 + WT-β1 (**A**) and with Nav1.5 + E87Q-β1. (**B**) Merged images (right panels in **A** and **B**) display the α and β1 NaCh subunits co-localisation in yellow. The scale bar is 20 μm. The distribution of β1 and Nav1.5 subunit fluorescence in Nav1.5 + WT-β1 (**C**) and Nav1.5 + E87Q-β1 (**D**) transfected cells was determined by comparing red and green fluorescence along a line drawn across a cell, as shown in the inset of the panels.

To confirm the biochemical analysis, we investigated the expression of Nav1.5 and β1 NaCh proteins by immunofluorescence (Fig. 6). Co-expression of WT-β1 with Nav1.5 generated an undistinguishable fluorescence pattern, particularly at the level of the cell membrane (Fig. 6A right panel and 6C), indicating a strict co-localization of both subunits. On the contrary, co-expression of the mutant E87Q-β1 with Nav1.5 resulted to a poorer co-localization, as turned out when merging the fluorescence signals (Fig. 6B right panel and 6D).

## Discussion

We accomplished the biophysical and biochemical characterization of the mutation E87Q of the NaCh β1 subunit. This mutation is linked to the Brugada syndrome 5 (OMIM 612838), a hereditary disease responsible for ventricular fibrillation, right precordial ST segment elevation on ECG and sudden cardiac death in the young. Our electrophysiological characterization confirms the results obtained by a previous study showing that mutation E87Q modifies NaCh functional expression when heterologously expressed in mammalian cells^29^. Our results clearly show that the primary defect of E87Q mutation of the β1 subunit is to compromise the correct NaCh maturation and traffic.

The most salient effect of the heterologous co-expression of the WT-β1 subunit with the pore-forming α subunit is to augment the functional expression of the NaCh, resulting in an increase of sodium current density^5, 7, 10, 24, 31–35^. Conversely, co-transfection of the cardiac Nav1.5 α subunit with mutant E87Q-β1 yields a sodium current level similar to that of cells transfected with Nav1.5 alone (Fig. 1A–E). These data are in agreement with the increase of Nav1.5 protein expression observed when cardiac NaCh α subunit is co-transfected with the WT-β1 subunit, whereas Nav1.5 protein expression is quite reduced when Na1.5 is co-expressed with the mutant E87Q-β1subunit (Fig. 2D). On the basis of these observations, we could argue that mutation E87Q of the ancillary subunit may reduce the capability of the β1 subunit to drive the expression of the NaCh complex into the plasma membrane for reviews see ref. 36 (Fig. 1F and Table 1).

Beside the influence of the β1 subunit on the docking of the NaCh at the plasma membrane, it also slightly modifies the activation and inactivation steady-state voltage-dependence. Co-expression of WT-β1 produces a negative shift of both, activation and inactivation, when compared with Nav1.5 alone, while the mutant E87Q-β1 is capable to shift only the voltage-dependence of inactivation, (Fig. 1F and Table 1). These data reinforce the hypothesis that the interaction between the α and the E87Q-β1 subints probably persists, but the mutation reduces the efficency of the β1 to favour the NaCH protein traffic. The E87Q mutation localizes into the extracellular Ig fold of the β1 protein, which contains critical charged residues that interact with the α subunit, and modulate its activity^6, 23^. The substitution of a charged glutamic residue with a glutamine in the Ig fold of the β1 protein, may produce a destabilization of this region, modifying the effectiveness of the interaction between the two NaCh subunits.

We explored the protein expression of each NaCh subunit in cells transfected with Nav1.5, Nav1.5 + WT-β1 and Nav1.5 + E87Q-β1. The presence of the RNA transcripts of both NaCh subunits was controlled in each preparation (see supplementary data). The RNA coding for Nav1.5 α subunit was about constant in all preparations, independently of the expression (or not) of either the WT- or the mutant β1 subunit. Similarly, the relative concentrations of the RNA transcripts of WT- or E87Q-β1 subunit were comparable. Therefore, any effect on NaCh expression could be considered a post-transcriptional phenomenon. Glycosylation is among the most relevant post-translational modifications on membrane proteins, that influences the docking of the protein to their final location in membrane. Indeed, glycosylation of the NaCh has been demonstrated to be pivotal for the channel targeting^37, 38^.

When we evaluated the expression of NaCh proteins in transfected cells, both Nav1.5 and β1 proteins in cell lysates, we detected two electrophoretic bands of slightly different molecular mass (Fig. 2A and B). We interpreted these bands as corresponding to different glycosylation and, consequentely, maturation states of the NaCh proteins. The first band, of lower molecular mass, may correspond to an immature protein with few or no post-translational modifications. This fraction of protein, that is only partially glycosylated, is probably waiting to be processed. Instead, the second band, of a higher molecular mass, should correspond to the mature polypeptide that has completely undergone the process of glycosylation^30, 37–40^. This fully glycosylated protein is going to be trafficked to the plasma membrane or has already reached it. It is interesting to notice that neither the expression nor the maturation of the auxiliary subunit are influenced by the E87Q mutation. In fact, not only the expression levels of total WT- and E87Q-β1 are similar, but also the fractions of the two proteins, the mature, glycosylated, and the immature, partially glycosylated, are not significantly different (Fig. 2A).

The analysis of the Nav1.5 protein expression shows that the mature, glycosylated, fraction of Nav1.5 is dramatically increased by the co-expression of WT-β1, while E87Q-β1 does not modify the fraction of mature Nav1.5 with respect to Nav1.5 alone (Fig. 2D, central panel). Conversely, both β1 subunit isoforms seem to not affect the level of the immature Nav1.5 fraction. Therefore, the increase of the total Nav1.5 expression, and probably its correlation to the increase of sodium current density promoted by the WT-β1 is due to an increase of the mature fraction of the α subunit. The mutated β1 subunit appears to lose this capacity to increase the expression of mature Nav1.5 (Fig. 3).

Biotinylation experiments, that reveal the fraction of protein in the plasma membrane, confirmed that co-expression of WT-β1 determines an increase of mature, fully glycosylated Nav1.5. On the contrary co-expression of E87Q-β1 with Nav1.5 does not modify the expression of the fully glycosylated Nav1.5 (Fig. 4). This evidence underlines how the maturation of Nav1.5 α subunit strongly depends on the presence of a counterpart, the auxiliary β1, capable not only to associate with the pore-forming α subunit and promote its maturation but also to promote its traffic to the cell membrane. These data were confirmed by the immunofluorescence experiments. It is appreciable a good co-localization of Nav1.5 and WT-β1 in correspondence of the cell boundaries (Fig. 6A and C). Presumably this co-localization regards the NaCh proteins that are undergoing the glycosylation process into the ER as well as those proteins that have already reached the plasma membrane. Co-localization resulted dramatically decreased in cells co-expressing Nav1.5 and E87Q-β1 (Fig. 6B and D), confirming that the E87Q mutation may reduce the β1 capability to interact with the α subunit.

The NaCh β1 subunit exerts two different actions: one on the traffic of the channel to the membrane, and the second on the modulation of the channel function. Data clearly demonstrate that the E87Q mutation significantly reduces, without completely abolishing, the capability of the β1 subunit to favour the docking of the α Nav1.5 subunit to the membrane. On the other hand, the action of the β1 subunit on the voltage-dependent properties of the sodium channel results more subtle, and even subjected to controversies^6, 32, 41^. In fact, the β1 subunit significantly changes the voltage dependence of the channel, but the mutation produces just a small variation of the activation curve. Therefore, we conclude that the interaction between the α and the E87Q-β1 NaCh subunits probably persists, but the mutation reduces the efficiency of the β1 to favour the protein traffic.

Under this perspective, it is to note that BrS exhibits an autosomal dominant pattern of transmission and variable penetrance^42^. Affected individuals could have one copy of mutant *SCN1B* and one of normal *SCN1B*. One way in which the E87Q mutation could exert its detrimental effects is if the mutant protein acts as a competing subunit by binding to the cardiac α subunit and preventing its association with the WT-β1, impeding Nav1.5 maturation into a functional NaCh. Also C121W, the best characterized β1 epileptogenic mutation, seems to behave as a competitor of WT-β1, modifying, without completely abolishing, the activity of normal NaCh^10, 24, 33, 43^. Our findings suggest that BrS type5 could be due to a reduced functional expression of the NaCh on the plasma membrane caused by an impaired interaction between the two subunits that constitute the channel. A reduced availability of NaChs on the cell surface, together with the alteration of the NaCh activation curve, would lead to the destabilization of cardiac cell excitability, predisposing the individuals affected by the E87Q mutation to ventricular fibrillation, a distinctive feature of BrS^44, 45^. This mechanism has been also described for other SCN5A BrS-related mutations^44, 46–53^. A reminiscent question remains still open: although the β1 subunit is ubiquitously expressed, mutation affecting this NaCh subunit are selectively associated with CNS or heart pathologies (for a review see ref. 9). The extension of our studies on the effect E87Q mutation on the expression of CNS sodium channel subtypes would be helpful for the elucidation of the molecular mechanisms underlying the association of β1 mutations with tissue-specific diseases.

## Conclusions

In summary, our results show that the role of β1 subunit on the maturation and expression of the entire NaCh complex is of paramount importance for the pathological conditions resulted from a defective interaction between the NaCh constituents. The present study demonstrates that mutation E87Q produces a modification of NaCh activation properties as well as a reduction of the expression of the NaCh on the plasma membrane. This is congruent with a reduced capability of the mutant β1 subunit to associate with the pore-forming α subunit and favour its translocation toward the plasma membrane. A more complete comprehension of the physiopathology of the defect will require to accomplish additional analysis in native tissues.

The behaviour of E87Q-β1 subunit ascribes this mutant subunit to the group of proteins responsible of diseases caused by defects in protein trafficking, as cystic fibrosis, lysosomal storage diseases (Fabry, Gaucher, and Tay-Sachs), nephrogenic diabetes insipidus, oculocutaneous albinism, protein C deficiency, and many others for a review see ref. 54. This characteristic suggests the possibility to enforce pharmacological interventions (pharmacological or chemical chaperones) to promote the rescue of the mutant protein to ameliorate the trafficking of the protein and therefore attenuate the symptoms of the disease.

## Material and Methods

### Cell culture

Chinese hamster ovary (CHO) cells were grown in Ham’s F10 medium supplemented with 2 mM L-glutamine and 10% FBS, at 37 °C and 5% CO_2_. To prevent the loss of differentiation potential, cells were not allowed to become confluent.

### DNA constructs and transfection

The pRcCMV plasmid construct containing the cDNA codifying for the human Nav1.5 pore forming subunit was a gift from Jean-Francois Desaphy (Università degli Studi di Bari, Dept. Pharmacy & Drug Sciences). pRcCMV plasmid construct containing the cDNA codifying for the human sodium channel ancillary β1 subunit was a gift from Alfred L. George (Division of Genetic Medicine, Vanderbilt University School of Medicine, Nashville, TN, USA). Mutant E87Q of the β1 subunit was obtained using the QuickChange kit (Stratagene, Santa Clara, USA), according to the manufacturer’s instructions. The mutation was verified by DNA-sequencing (Biofab Research, Rome, Italy). For transfection, CHO cells were plated onto poly-L-lysine-coated culture dishes and grown to 50% confluence in complete medium. Cells were transiently transfected using Lipofectamine 2000 (Invitrogen, Paisley, UK) with 1 μg of Nav1.5 cDNA and 1 μg of β1 subunit cDNAs, and used between 48 and 72 h after transfection. Efficiency of transfections was evaluated by immunofluorescence (see Supplementary Information).

For the electrophysiological measurements, cells were co-transfected with 50 ng of pcI-CD8 cDNA and the success of transfection was tested using CD8-antigen coated microspheres (Dynabeads Dynal, Invitrogen, USA). Transient expression was tested electrophysiologically between 48 and 72 h after transfection. Only cells that showed the expression of CD8 receptor by capturing the CD8-antigen covered microspheres were used for the electrophysiological experiments.

In experiments where Nav1.5 was transfected with different molar ratios of WT- and E87Q- β1 subunits, the total amount of β1 subunit cDNAs was 1 μg.

### RNA isolation, reverse transcription and real time quantitative PCR

Total RNA was isolated using the RNeasy Mini kit (Qiagen, Hilden, Germany) and first-strand cDNA was synthesized from 2 µg of RNA using the RevertAid First Strand cDNA Synthesis Kit and random hexamers according to the manufacturer’s instructions (Fermentas, Burlington, Canada). First-strand cDNA from transfected CHO cells was employed as the template in real-time polymerase chain reaction (PCR) amplifications using pairs of oligonucleotide primers specific for the human Nav1.5 and β1 subunits and amplification conditions as described elsewhere^8, 55^. Glyceraldehyde-3-phosphate-dehydrogenase (GAPDH) was used as a reference gene. Sequence of the PCR-primers are detailed in the Supplementary Information. Changes in cDNA amount were quantified by real-time PCR (CFX Connect™ Real-Time PCR Detection System instrument, Bio-Rad Laboratories, Hercules, CA, USA) by using the comparative Ct method. Each sample was run in triplicate.

### Electrophysiological measurements

Sodium currents were measured with the patch clamp technique in the whole cell configuration using an Axopatch 200B amplifier (Axon Instruments, Foster City, CA, USA). Borosilicate glass micropipettes were fire polished yielding a resistance from 1.5 to 2.5 MΩ with the working solutions. The pipette was filled with (in mM): 20 NaCl, 120 CsF, 2 EGTA, 10 HEPES buffer at pH 7.3. The external solution had the following composition (in mM): 145 NaCl, 2.5 KCl, 1 MgCl_2_, 2 CaCl_2_, 10 Glucose, 10 HEPES at pH 7.3. The output of the patch clamp amplifier was filtered by the low-pass 4-pole Bessel filter with a cut-off frequency of 10 kHz and sampled at 50 kHz. The cell was kept at a holding potential of −120 mV. Pulse stimulation and data acquisition used 16 bit D-A and A-D converters (NI PCI-6221, National Instruments, Austin, TX, USA) controlled by a PC with a custom acquisition program (Gepulse, users.ge.ibf.cnr.it/pusch/programsmik.htm). The remaining linear responses after analogical compensation were digitally subtracted with a standard P/4 protocol. Access resistance was always less than 8 MΩ, and series-resistance was carefully compensated (between 80 and 95%). All measurements were done at room temperature (21 ± 1 °C). Electrophysiological data were analysed using a custom procedures developed under IgorPro (Wavemetrics, Lake Oswego, OR, USA). The voltage dependence for the channel activation was evaluated by fitting the peak sodium current, *I*_*peak*_, elicited by a different test pulse potentials *V*, using the equation:

1 $${I}_{peak}(V)={\rm{\Gamma }}\frac{1}{1+\exp [(V-{V}_{1/2})/{S}_{a}]}(V-{V}_{rev})$$

where Γ is the maximum sodium conductance, *V*_1/2_ is the half activation potential, *S*_*a*_ is the slope of the function, and *V*_*rev*_ is the reversal potential. To determine the voltage dependence of the steady-state inactivation, the sodium current was elicited by a fixed-amplitude test pulse preceded by a conditioning 100 ms pulse. The test pulse peak current, *I*_*test*_, was plotted against the prepulse potential, *V*_pp_, and fitted with:

2 $$\frac{{{1}}_{test}}{{{1}}_{{\max }}}=\frac{1}{1+\exp [-{V}_{pp}-{V}_{h})/{S}_{h}]}$$

where *I*_*max*_ is the maximum current, *V*_*h*_ is the half inactivation potential and *S*_*h*_ is the voltage dependence of inactivation. The recovery from inactivation was determined with a double pulse protocol, with a 10 ms pre-pulse to −20 mV to evoke the maximal inactivation, followed by a variable recovery interval (0.5 to 40 ms) of a hyperpolarized potential of −120 mV. The inactivation-recovered channels were then measured by a test pulse of −10 mV. Time constants of recovery from inactivation were estimated by fitting peak current at test pulse against the recovery time interval to a single exponential function.

### Western blot

Cells were lysed in a buffer containing 62.5 mM trisaminomethane (TRIS), 2% Sodium dodecyl sulfate (SDS) and a cocktail of protease inhibitors (1 mM 4-(2-Aminoethyl) benzenesulfonyl fluoride hydrochloride-AEBSF, 0.8 μM Aprotinin, 0.2 μM Leupeptin, 40 μM Bestatin, 15 μM Pepstatin A, 14 μM E-64). Protein concentration was determined using the method of Lowry^56^ with bovine serum albumin as the standard. Equal amounts of proteins (40 μg) were subjected to SDS poly-acrylamide gel electrophoresiss. Separated proteins were transferred to PVDF membrane (Millipore) for 1 h at 100 V. The blots were then incubated with polyclonal rabbit anti-Nav1.5 (1:200) or anti-β1 (1:500, Abnova, Taipei city, Taiwan) as primary antibodies, and with horseradish peroxidase-conjugated goat anti-rabbit antibody (1:5000), as secondary antibody. Immunodetection was performed using Amersham ECL PLUS detection reagents (GE Healthcare, Marlborough, MA, USA) and the images were captured by using Amersham Hyperfilm ECL (GE Healthcare). Developed films (Kodak, Rochester, NY, USA) were scanned using a flat-bed scanner with a resolution of 1200 dpi. The intensity of the electrophoretic bands was quantified from digital images using a custom procedure developed under IgorPro. In order to confirm the homogeneity of the loaded proteins, immunoblots were stripped by incubating them in the a buffer containing 62.5 mM TRIS pH 6.8, 10% SDS, and 1% β-mercaptoethanol for 30 min at 55 °C, and re-probed with a polyclonal anti-actin primary antibody (1:3000, Sigma Aldrich). The intensity of each band was normalized to the intensity of the band corresponding to the actin detected in the stripped PVDF membrane.

### Deglycosylation assay

For deglycosylation experiments, protein deglycosylation Mix II (New England Biolabs, Ipswich, MA, USA) was used according to the manifacturer’s instructions with some modifications. Deglycosylation Mix Buffer 2 (10 µl) was added to total cell lysates from transiently transfected Nav1.5, WT- and E87Q-β1 CHO cells (100 µg), respectively. Samples were incubated at 75 °C for 10 min. After cooling on ice, denaturated proteins were supplemented with Protein Deglycosylation Mix II (10 µl) and incubated at room temperature for 30 minutes. Samples were finally incubated at 37 °C for 3 hours. The extent of Nav1.5 and β1 deglycosylation was assessed by SDS-PAGE and Western blot analysis.

### Surface biotinylation

Cells transfected with the sole Nav1.5, Nav 1.5 + WT-β1, or Nav1.5 + E87Q-β1 were grown in four T75 flasks; membrane proteins were biotinylated using the Pierce Cell Surface Protein Isolation Kit (Thermo Fisher Scientific, Waltham, MA, USA) following the manufacturer’s instructions. Briefly, intact cells were washed once with ice-cold PBS, resuspended at a concentration of 10^7^ cells/ml in ice-cold biotinylation mix (12 mg NHS-SS-biotin dissolved in 12 ml ice-cold PBS, pH 8.0), and incubated for 30 min at 4 °C. Biotinylation of cell surface proteins was quenched by the addition of 1 ml quenching solution (Thermo Fisher Scientific). Labeled cells were washed twice in ice-cold TRIS-buffered saline, pH 7.4, and centrifuged at 500 × g for 3 minutes. The pellet was dispersed in 100 μl 50 mM TRIS-HCl, pH 7.6, using the plunger of a 1-ml syringe with a 36 gauge needle, and the preparation was made up to a final volume of 500 μl lysis buffer at a final concentration of 0.5% (w/v) SDS, 1% (v/v) Triton, 150 mM NaCl, 1 mM EDTA, 10 mM Tris-HCl, protease inhibitor cocktail, and 0.1 mg/ml PMSF. Samples were ultra-sonicated for 30 s at medium power (UP200Ht, Hielscher Ultrasonics, Teltow, Germany) at 4 °C, and the lysates were centrifuged for 10 min at 16,000 g. Biotinylated cell surface proteins were enriched using 500 μl Neutravidin agarose beads (Thermo Fisher Scientific) for 2 h at 4 °C. Beads were washed three times with lysis buffer, then twice with lysis buffer containing 500 mM NaCl, and then once with salt-free lysis buffer. Biotinylated cell surface proteins were eluted from beads by incubation for 1 h at room temperature in 1% (w/v) SDS, 50 mM DTT, 100 mM Tris-HCl. Proteins were denatured subsequently by heating for 20 min at 50 °C. Samples were loaded on SDS-PAGE gel and processed for Western blot. Quantified immunoreactive signals were normalized to cadherin, a cell surface housekeeping protein. Biotinylated and cell lysate proteins from transiently transfected Nav1.5, WT- and E87Q-β1 CHO samples were also probed with anti GM130 primary antibody (1:200, BD Biosciences, San Josè, CA, USA), which is a cis-Golgi marker^57^.

### Detection of Nav1.5 and β1 subunits by immunofluorescence

Transiently transfected Nav1.5, WT- and E87Q-β1 CHO cells were immunofluorescence stained in order to examine the expression efficiency of each single construct. Cells were fixed with 4% paraformaldehyde and washed 3 times with PBS before use. Cells were permeabilized in PBS, 0.3% Triton X-100, and 10% normal goat serum, and incubated overnight with polyclonal rabbit anti-Nav1.5 (1:200, Millipore, Billerica, Massachusetts, USA) or goat anti-β1 (1:500; Santa Cruz biotechnologies, Santa Cruz, CA, USA) primary antibodies diluted in PBS and 10% normal goat serum. After primary antibody incubation, cells were incubated for 2 h in goat anti-rabbit secondary antibody coupled to fluorescein isothiocyanate for the detection of the Nav1.5, and in donkey anti-goat coupled to tetramethylrhodamine for the detection of the β1 subunit. Samples were washed three times with PBS after each antibody step. Micrographies were collected using a laser scanning spectral confocal microscope (TCS SP2-AOBS; Leica Microsystems, Heidelberg, Germany). Image analysis was performed using Leica and ImageJ^58^ softwares.

### Statistics

All data are given as mean ± standard error of the mean (sem). Statistical significance of differences among mean values was assessed by using Student’s t-test. Differences were regarded as statistically significant for a probability, p < 0.05.

### Chemicals

Except when indicated, all reagents were purchased from Sigma Aldrich (Milano, Italy).

## Electronic supplementary material

Supplementary materials
