# Supplementary material for: Mutation E87Q of the β1-subunit impairs the maturation of the cardiac voltage-dependent sodium channel
Source: Sci Rep. 2017 Sep 6;7:10683. doi: 10.1038/s41598-017-10645-y (PMC5587543; doi:10.1038/s41598-017-10645-y)
Supplement: Supplementary file 1 — Supplementary materials [file 41598_2017_10645_MOESM1_ESM.pdf]

Mutation E87Q of the β1-subunit impairs the maturation of the cardiac voltage-dependent sodium channel

Debora Baroni, Cristiana Picco and Oscar Moran

Supplemental materials

1. Evaluation of transfection efficiency

The efficiency of transfection of vector constructs containing the cDNAs codifying for Nav 1.5, WT-β1 or E87Q-β1 NaCh subunits was evaluated by immunofluorescence. We estimated the transfection efficiency of Nav1.5 α subunit transfected alone, or co-transfected with WT- or E87Q-β1 subunits. Evaluation of the transfection efficiency was done in at least three independent preparations, observing >1000 cells for each condition. The percentage of transfected cells was 63 ± 1%, 66 ± 1%, and 62 ± 2%, in CHO cell preparations transiently transfected with Nav 1.5 alone, with Nav1.5 and WT-β1 or with Nav1.5 and E87Q-β1, respectively. Figure S1 shows an image of CHO cells trasfected with the Nav1.5 cDNA construct. The WT- and E87Q-β1 cDNA construct transfection efficiency was 65 ± 1%, and 62 ± 2% in CHO cell preparations transfected with Nav1.5 and WT-β1 and with Nav1.5 and E87Q-β1, respectively. As retrieved data were not statistically different, it was not necessary to proceed to any further correction of the results of mRNA and protein expression to the transfection efficiency, considering that data have been also normalised to the expression of a housekeeping gene. In untransfected CHO cells, the antibodies raised against β1 or Nav1.5 subunits did not reveal the presence of these NaCh subunits.

A **B**

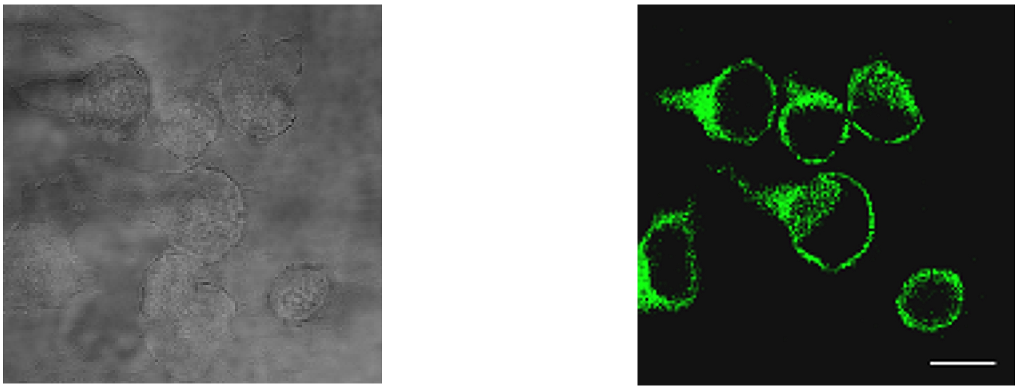

Figure S1. *Evaluation of sodium channel Nav1.5 subunit cDNA construct transfection efficiency by immunofluorescence.* Representative images showing the light transmision (**A**), and the fluorescence micrographs (**B**) of CHO cells transientely transfected with the Nav1.5 (green) subunit cDNA construct. The scale bar is 20 μm.

2. Expression of sodium channel subunit mRNAs.

The relative abundance of each NaCh subunit mRNA was evaluated by real-time PCR. Figure S2 represents the β1 and Nav1.5 subunit mRNA levels revealed in Nav 1.5, Nav1.5 + WT-β1 and in Nav1.5 + E87Q-β1 CHO transfected cells using primer sets specific for the two NaCh subunits Table S1). The expression level of the mRNA coding for the β1 ancillary subunit resulted absent in untransfected and in cells transfected with only the Nav1.5 cDNA construct and was similar in cells transfected with Nav1.5 + WT- and with Nav1.5 + E87Q-β1 (1.00 ± 0.03 and 1.04 ± 0.03, respectively). Analogously, the relative abundance of Nav1.5 mRNA in CHO cells transfected with Nav1.5 alone (1.02 ± 0.06) was not statistically different from that of cells transfected with Nav1.5 + WT-β1 or with Nav1.5 + E87Q-β1 (1.02 ± 0.05 and 1.01 ± 0.06, respectively). The mRNA codifying for Nav1.5 NaCh subunits was not detected in untransfected CHO cells.

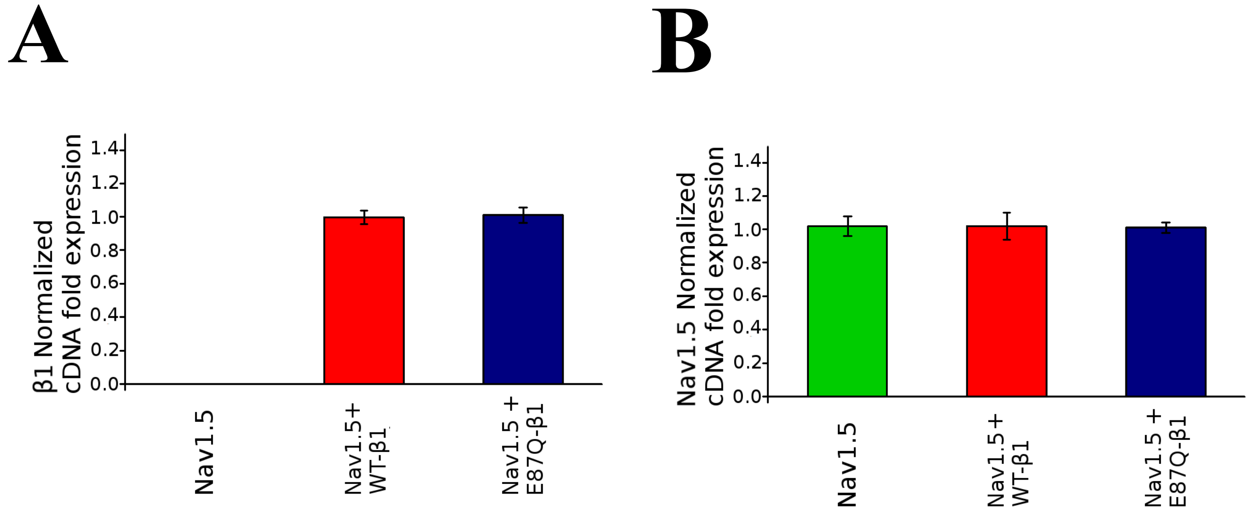

FigureS2. *Detection of transcripts of sodium channel β1 and Nav1.5 subunits in CHO transfected cells*. (**A**) Detection of transcripts of sodium channel β1 subunit in Nav 1.5, Nav1.5 + WT-β1 and in Nav1.5 + E87Q-β1 CHO transfected cells. (**B**) Detection of NaCh Nav1.5 subunit transcripts in Nav 1.5, Nav1.5 + WT-β1 and in Nav1.5 + E87Q-β1 CHO transfected cells. Transcripts of β1 and Nav1.5 subunits were revealed with specific oliguonucleotide primers. Bars represent the averaged fold change of β1 and Nav1.5 subunit mRNA expression in transfected CHO cells normalized to Glyceraldehyde-3-phosphate-dehydrogenase (GAPDH) used as housekeeping gene. Each experiment was done in triplicate. Asterisks (*) indicate a significant difference ($p < 0.05$) in the comparison with the transcript level of level either of β1 or Nav1.5 in Nav1.5 + β1 CHO transfected cells.

Table S1. *Primers employed in real time-PCR experiments*

target	forward primer	reverse primer
Nav1.5	5'-GCTACACCAGCTTCGATTCC-3'	5'-GGGTGAGGCTGAGATGATTC-3'
β1 subunit	5'-CTTCAGACACGCACTTCTGG-3'	5'-TTCAAGGCTGGTGAGAGAGG-3
GAPDH	5'-CAAGGTCATCCATGACAACTTTG-3'	5'-GTCCACCACCCTGTTGCTGTAG-3'

3. Analysis of gel images

Luminiscence was detected on Amersham Hyperfilm ECL (GE Healthcare). Developed films were scanned using a flat-bed scanner Agfa Snapscan Touch e26 with a resolution of 1200 dpi. The intensity of the electrophoretic bands was quantified from digital images using a custom procedure developed with the data analysis program suite IgorPro. There, a region of interest is selected and the optic density profile is obtained (Figure S3). The densitometric curves were fitted with a Gaussian function with a constant background. When two near peaks were detected, data were fitted with the summatory of Gaussian functions. Bands whose signal resulted saturated (see insert in figure S3) were discarded for further analysis.

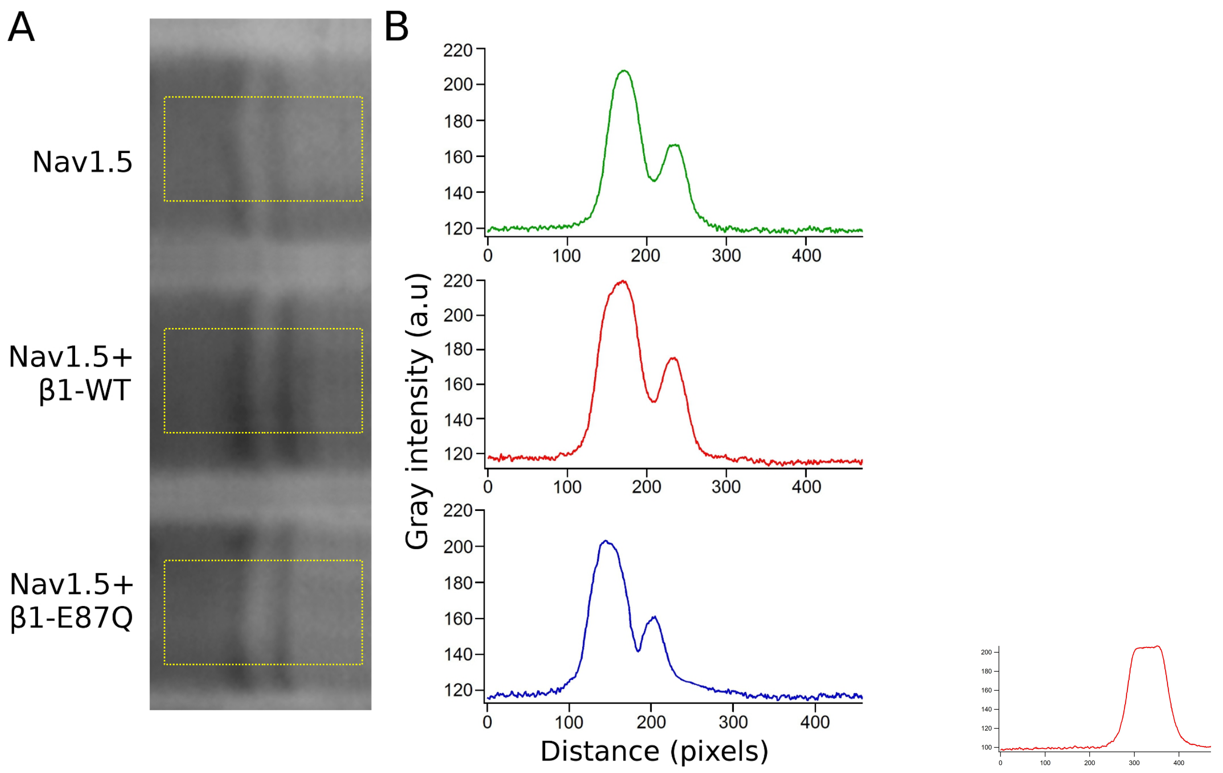

Figure S3. SDS-PAGE and Densitometry analysis of Nav1.5 expression in Nav 1.5, Nav1.5 + WT-β1 and in Nav1.5 + E87Q-β1 CHO transfected cells. (**A**) The Western blot was probed with rabbit anti-Nav1.5 followed by horseradish peroxidase-conjugated goat anti-rabbit antibody. Immunodetection was performed using ECL PLUS detection reagents and images were developed by using ECL films. (**B**) Densitometry profiles for each lane of the Western blot shown in **A.** The insert on the right shows a densitometry profile of a saturated signal.

4. Western blot raw data

Data presented herein was digitised as described in the supplementary section 3. The gels in the experiments contained samples other than those included in the figures. The lanes presented on each figure are indicated in the corresponding image.

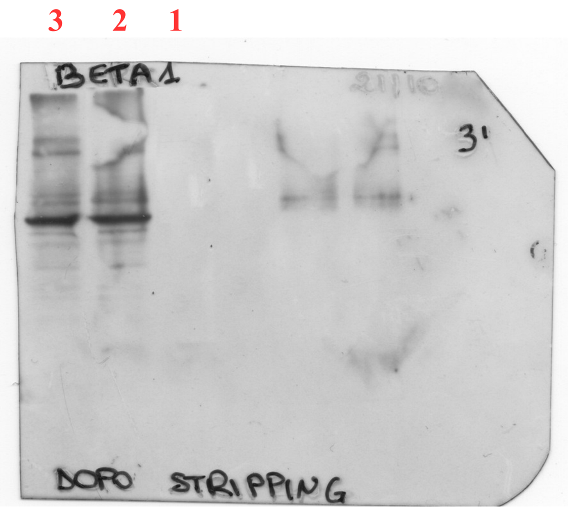

Figure 2A, β1. (whole cell lysates).
Notice that the order of the lanes was inverted in the figure

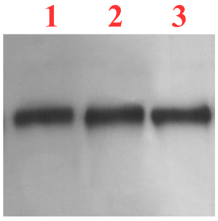

Figure 2A, Actin. (whole cell lysates).
Only the lanes corresponding to Nav1.5, Nav1.5 + WT-β1 and Nav1.5 + E87Q-β1 samples were originally acquired.

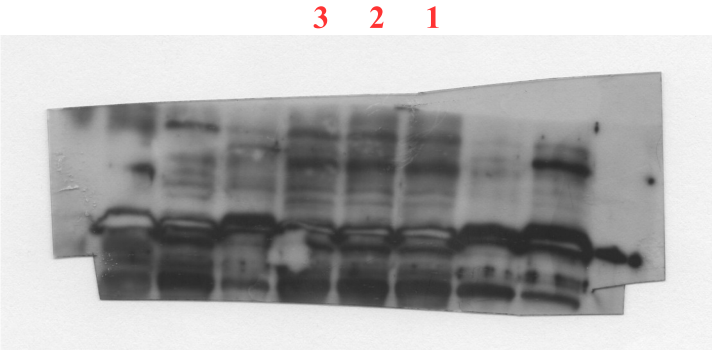

Figure 2C, Nav1.5. (whole cell lysates).
Notice that the order of the lanes was inverted in the figure.

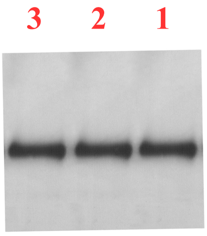

Figure 2C, Actin (whole cell lysates).
Only the lanes corresponding to Nav1.5, Nav1.5 + WT-β1 and Nav1.5 + E87Q-β1 samples were originally acquired.

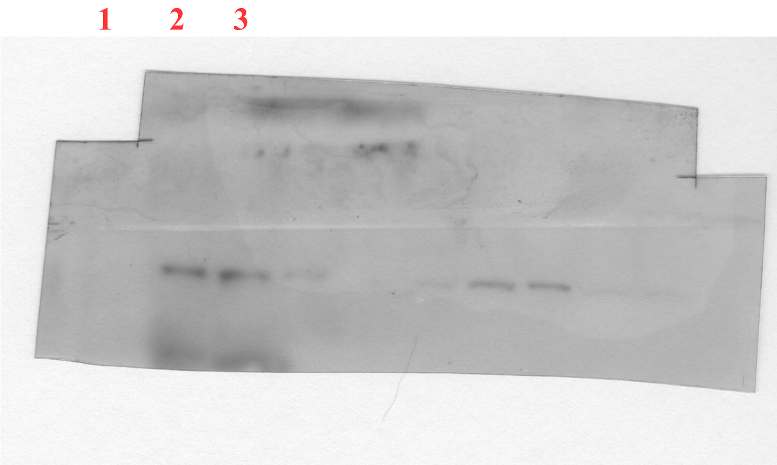

Figure 2E. β1 (deglycolysation)

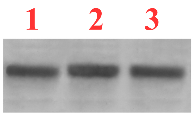

Figure 2E. Actin (deglycolysation).
Only the lanes corresponding to Nav1.5, Nav1.5 + WT-β1 and Nav1.5 + E87Q-β1 samples were originally acquired.

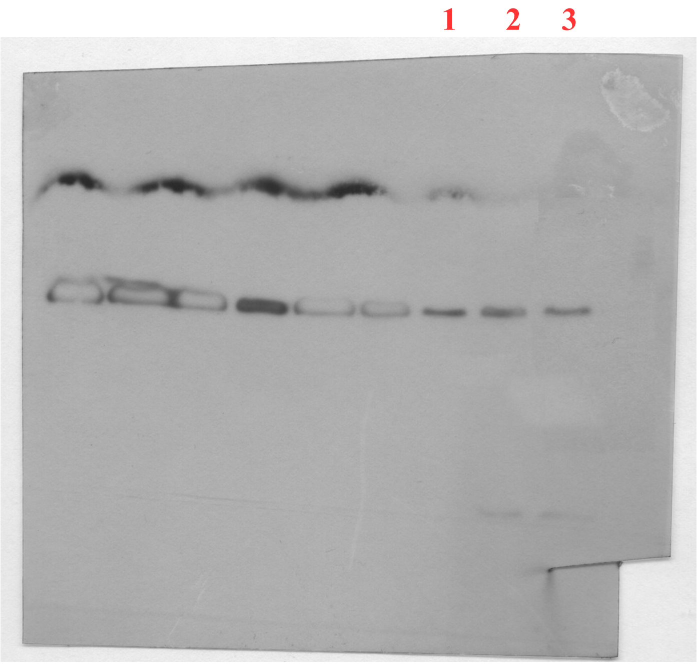

Figure 2E. Nav1.5 (deglycolysation)

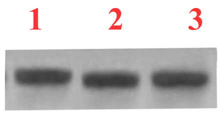

Figure 2E. Actin (deglycolysation).
Only the lanes corresponding to Nav1.5, Nav1.5 + WT-β1 and Nav1.5 + E87Q-β1 samples were originally acquired.

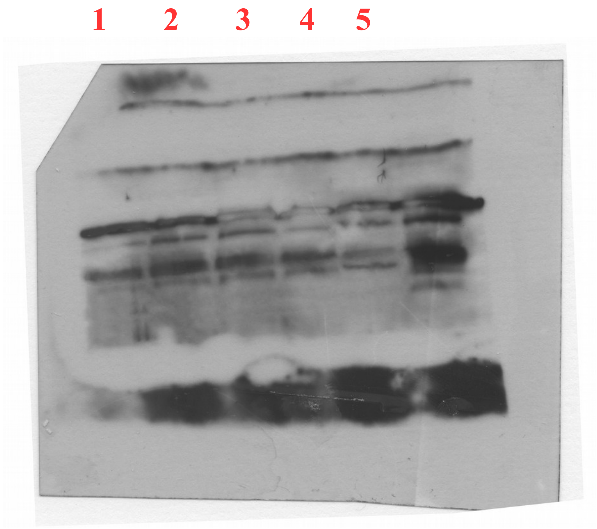

Figure 3A. Nav1.5

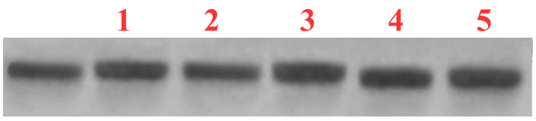

Figure 3B. Actin.
Only the lanes corresponding to Nav1.5, Nav1.5 + WT-β1 and Nav1.5 + E87Q-β1 samples were originally acquired.

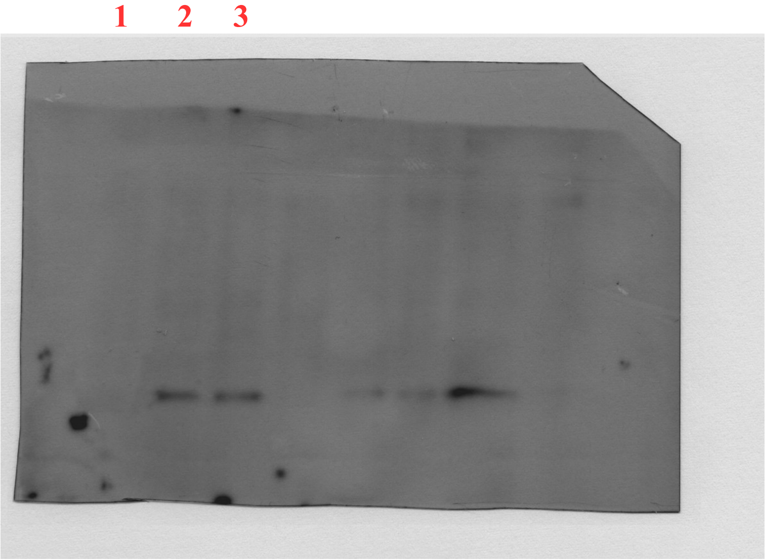

Figure 5A. β1 (Biotynilation)

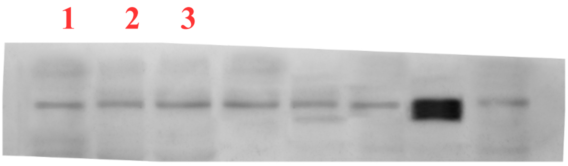

Figure 5A, Cadherin (Biotynilation).
Only the lanes corresponding to Nav1.5, Nav1.5 + WT-β1 and Nav1.5 + E87Q-β1 samples were originally acquired.

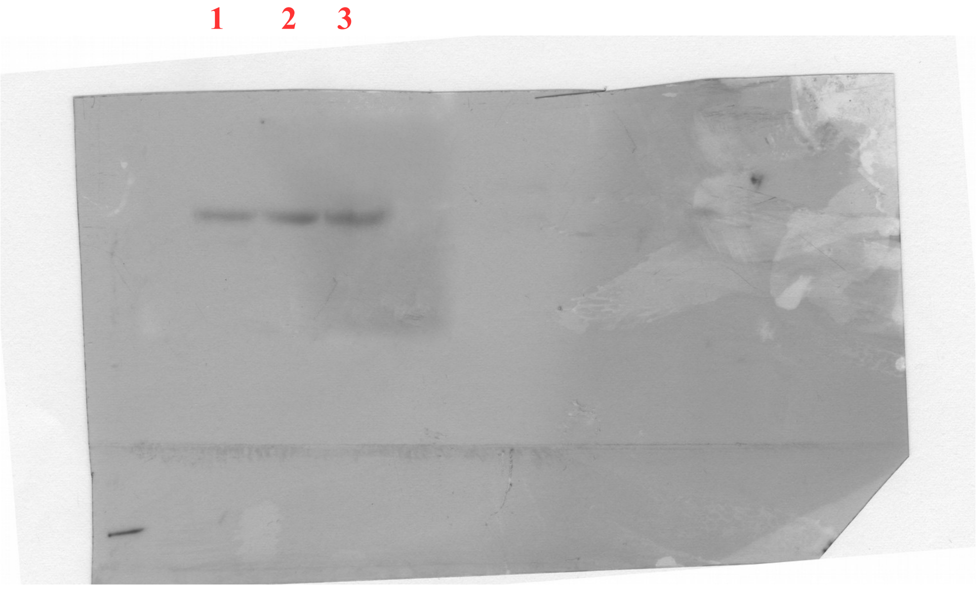

Figure 5B, Nav1.5 (Biotynilation)

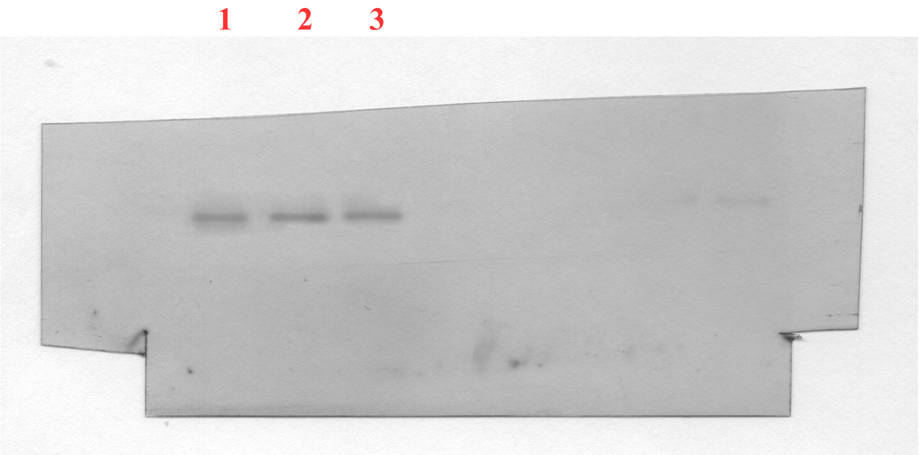

Figure 5B, Cadherin (Biotynilation)

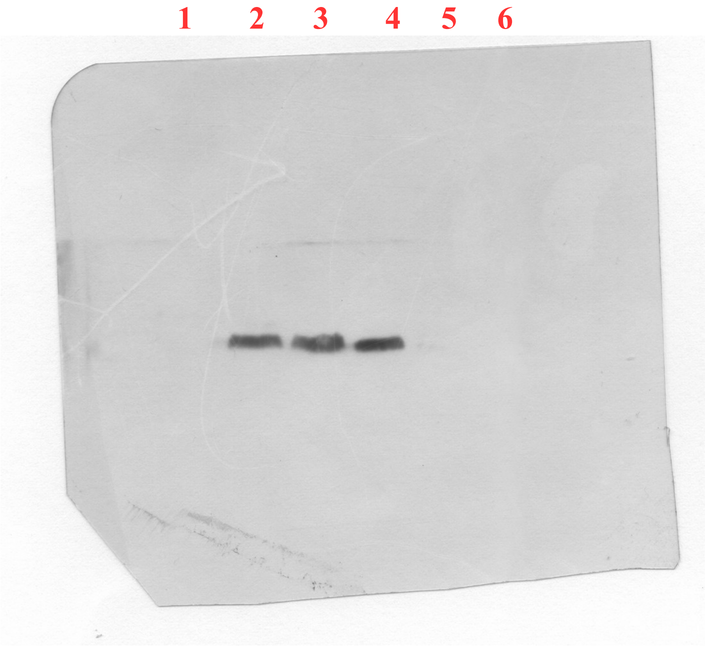

Figure 5C, GM130 (Biotynilation)
